# Human-elephant conflict in communities bordering Kruger National Park: Revealing causes and unpicking social media narratives

**DOI:** 10.1371/journal.pone.0350166

**Published:** 2026-07-08

**Authors:** Tom P. Moorhouse, Herbert Ntuli, Prince Nketiah, Angie Elwin, Suzi Paterson, Neil C. D’Cruze

**Affiliations:** 1 Oxford Wildlife Research, London, United Kingdom; 2 Department of Agricultural Economics, Extension and Rural Development, University of Pretoria, Hatfield, South Africa; 3 World Animal Protection, London, United Kingdom; University of Limpopo, SOUTH AFRICA

## Abstract

Conflict between humans and wildlife in communities living adjacent to protected areas is one of the most intractable issues facing policymakers and conservation practitioners. The presence of wildlife can negatively impact on humans in a number of different ways, from fatalities to loss of livelihoods. Proximity to human communities can also negatively impact wildlife, for example through retaliatory killings and illegal harvesting. Meeting conservation objectives therefore often requires measures to reduce human-wildlife conflict, especially at the borders of national parks. We examine the drivers of human-elephant conflict occurring in villages bordering Kruger National Park (KNP), South Africa – which can result in the killing of elephants that enter these communities. We use data from surveys in these communities, as well as online descriptions of an incident in the village of Matsulu which resulted in the death of an elephant and which was filmed by several community members, generating a large number of social media stories. Social media stories and survey responses both highlighted the same underlying drivers of elephant killings: that members of the community appear to have tampered with the KNP fence and encouraged elephants to exit the park. Community members benefited through obtaining elephant meat. Data from the survey additionally indicated that a small number of community members profit financially from elephant escapes, but that the wider community experiences a range of negative impacts, which may be partially defrayed by benefits in the form of meat. The majority (71.2%) of survey respondents stated they would support non-lethal elephant management approaches. The current policy regarding elephant management in KNP is that elephants are protected within the park but if outside and considered a danger to humans are permitted to be killed. This policy has implications for elephant conservation in a context where members of local communities may feel incentivised to facilitate the escape of elephants, in order to benefit from legitimately killing them. We recommend an evaluation of this policy in the light of community attitudes towards elephants and their management.

## Introduction

Conflict between humans and wildlife in local communities living adjacent to protected areas is one of the most widespread and intractable issues facing policymakers and conservation practitioners today [1]. Such conflicts can be variable, complex and multifaceted, with resultant external costs to both state agencies and park-adjacent communities [2,3,4]. As a result, local communities can have very different perceptions and attitudes towards losses from the presence of wildlife, which translate into different behaviours and degrees of willingness to live alongside a given species  [5].

The presence of wildlife can cause losses and trauma to humans in a number of different ways [1], e.g., through predation of livestock [6], crop-raiding or destruction of stored food [7, 8,9], direct attacks on humans [7,10,11,12], disease transmission to stock or humans [6], and costs from the restriction of life choices that can result from people’s proximity to conservation areas or the presence of wildlife [13]. Conversely, there is considerable potential for the presence of wildlife to benefit human populations [14], e.g., through biodiversity-related social compacts that aim to improve human wellbeing and social cohesion through reduced poverty and concomitant benefits, while securing the ecological systems on which life depends [15]. Such goals, however, require strategies that reduce trades-off between conservation and human wellbeing [15].

For wildlife populations, the close proximity of human communities can have a series of negative impacts [16], for example through retaliatory killings [17,18,19], and illegal harvesting and trafficking of valuable wildlife species both inside and outside of protected areas – for subsistence or commercial purposes [20], and as protest behaviour (in which individuals or groups express dissatisfaction with, or opposition to, a perceived injustice through peaceful demonstrations, petitions, boycotts or disruptive or confrontational actions such as poaching; [21,22,20]).The exploitation of wildlife for trade raises significant concerns due to its potential negative impacts on local ecosystems, species populations, and the well-being of indigenous communities whose livelihoods are dependent on the wildlife economy [23]. For all these reasons meeting conservation objectives often requires measures to reduce forms of human-wildlife conflict and consumptive uses, especially at the borders of national parks.

Human-wildlife interactions of local communities living adjacent to protected areas in Southern Africa can have disastrous consequences on both livelihoods and conservation outcomes [24,25,26]. In this study we examine human-wildlife conflict occurring in villages bordering Kruger National Park (KNP) in South Africa. In communities similar to these, benefits of wildlife to humans living adjacent to protected areas often take the form of some income generated through trophy hunting, both legally and illegally, and from the subsistence consumption and sale of wildlife-origin meat and other products [20,27].

**Fig 1:**
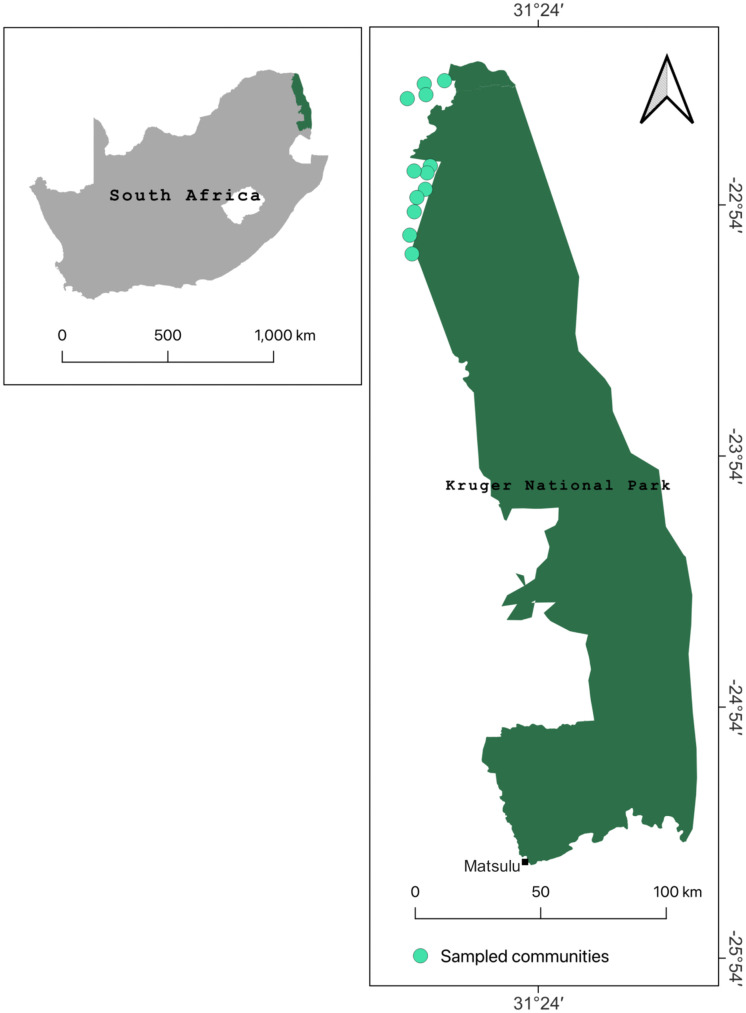
A map of the study area, showing the sampled communities to the north of Kruger National Park, and the position of Matsulu. Figure redrawn with permission from Moorhouse et al. [14].

One key, recent incident on the 10^th^ of February, 2024, involved an elephant that left KNP and entered the bordering community of Matsulu, a large township of 47,306 residents located in the Ehlanzeni District of Mpumalanga, South Africa, approximately 35 km west of the southern boundary of KNP (Fig 1). Matsulu’s position reflects apartheid-era spatial planning, which deliberately located Black African communities on the peripheries of urban areas and adjacent to national parks. Such positioning limited access to services, constrained infrastructure, and produced a reliance on communal resources, as well as increasing the risk of human-wildlife-conflict. In the specific case of human-wildlife conflict between elephants and communities surrounding KNP, local laws protect elephants when within the park, but once outside of the park boundary elephants are considered to be potential “damage causing animals”, which should be captured and returned to the park by local authorities [28]. Animals that represent a danger to human life are permitted to be destroyed by local authorities, or landowners (albeit with a requirement for further investigation in the latter case) [28].

In South Africa, the management of elephants and other threatened or protected species (TOPS) is governed by a combination of national and provincial frameworks. Within Kruger National Park (KNP), SANParks holds primary authority for wildlife management, while the Mpumalanga Tourism and Parks Agency (MTPA) oversees species and human–wildlife interactions in provincial areas [29,30]. The Damage Causing Animals (DCA) provisions outline procedures for managing problem elephants, including criteria for translocation (“herding back”) and euthanasia, applied when human life or property is at risk [31]. The South African Police Service (SAPS) may be involved in law enforcement or public safety measures, but routine wildlife decisions remain with SANParks or MTPA. Current policies may unintentionally shape incentives: DCA procedures may encourage rapid removal or lethal action rather than conflict prevention, while communities adjacent to parks may bear disproportionate risks without formal compensation.

Previous incidents of elephants entering communities have reportedly ended with elephants being returned to KNP [32], but the interaction that took place in the village of Matsulu resulted in the elephant being killed by the authorities, and in it being subsequently butchered for meat by the community – with the meat being distributed among local communities.

The interaction in Matsulu was notable for having been filmed by several community members, with a large number of social media stories being generated from the footage. Representations of human-wildlife interactions in social media videos and imagery, and the resultant public response, may influence public perceptions, societal behaviour and social norms regarding appropriate treatment of wild animals [33,34,35]. In the specific case of portrayals of interactions of local populations with African megafauna by both the editorial and social media, the narratives presented can contribute to social change [36]. As an example, global attention on the killing of Cecil the lion in 2015, particularly arising from social media, is credited with changing the nature of the debate around trophy hunting in many countries [37,38]. Similarly the killing of Harambe the gorilla at the Cincinnati Zoo (Ohio, United States) in 2016 sparked global outrage on social media, driving public debates about zoo safety, animal welfare, and management practices (e.g., [39,40]). While traditional media has historically not reported on issues that impact animals, social media stories often draw attention to individual animals, which in some cases have galvanized the global public and led to policy changes [41,42]. Public attention resulting from such social media stories, however, does not necessarily result in policy changes: analysis of the Cecil incident concluded that it did not elicit a large-scale change in policy, although it may have hastened policy reforms in some countries [43].

While social media portrayals of human-wildlife interactions can influence policy and spread awareness, they could also result in negative outcomes for wildlife. As an example, concerns are growing that displaying wild animal pets in the media could foster positive perceptions of their exploitation and increase demand for them, potentially fuelling the wildlife trade [44,45]. The tone and content of social media commentary on human-wildlife conflict is therefore a key determinant of the wider societal impacts of a given interaction, with the potential to change wider narratives for the benefit of species conservation and animals’ welfare, or to their detriment.

Here we present an overview and analysis of the Matsulu incident, as well as analysis of the attitudes to, and likely drivers of, human-elephant conflict in communities surrounding KNP. Our primary aim is to reveal the underlying causes of such incidents, anticipating that the information would underpin recommendations to inform and improve mechanisms for handling human-wildlife conflicts that arise from elephant encounters outside KNP. A secondary aim is to examine the dominant narratives in media descriptions of the Matsulu incident to determine the extent to which media accounts capture the drivers of the incident – and therefore to represent these drivers accurately to interested members of the public and shape discourse around similar events in the future.

## Methods

### Research design

**Fig 2:**
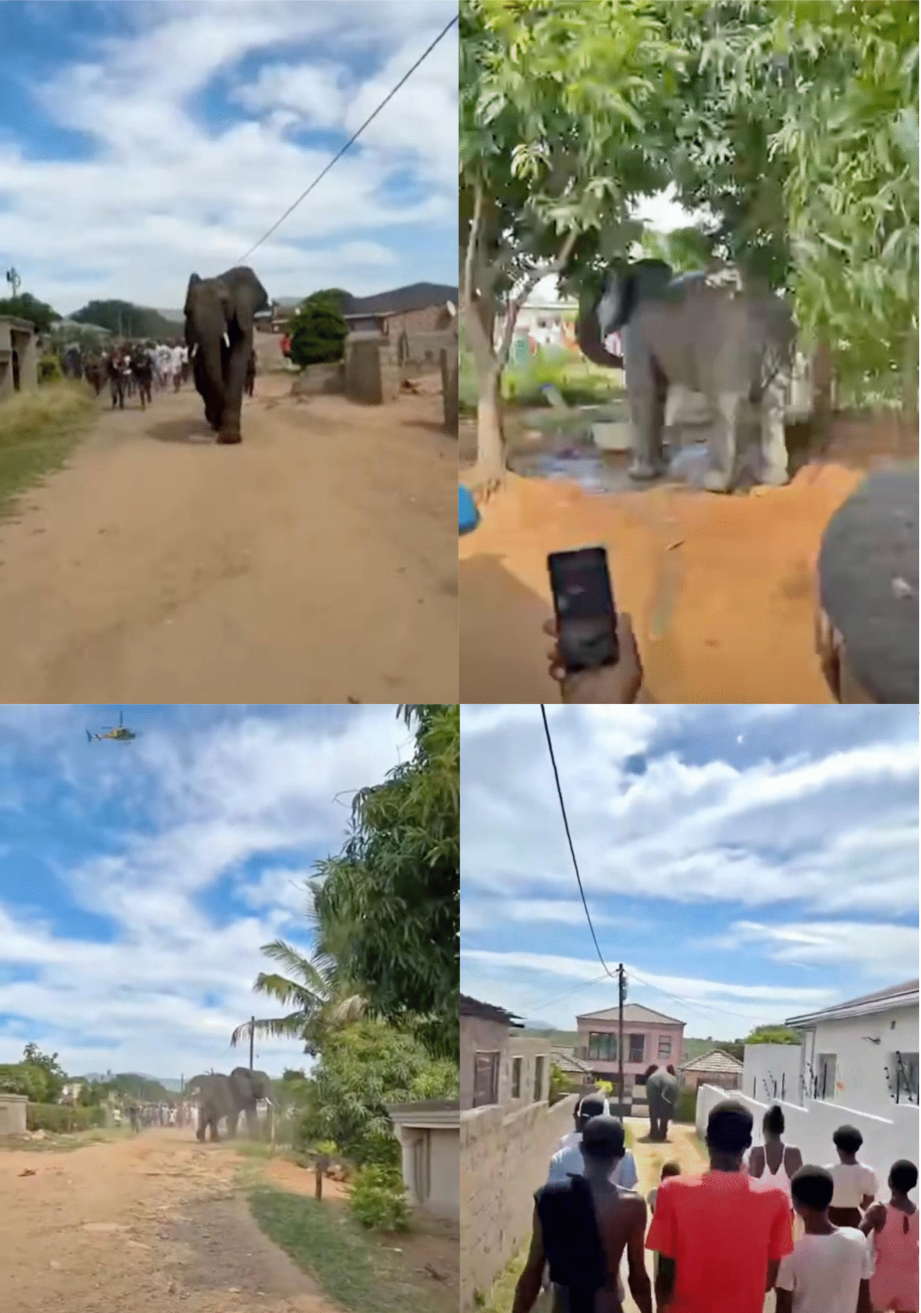
Illustrative social media still images from online videos of the Matsulu incident.

We employed mixed methods to address our study aims. First, we used descriptive statistics to analyse responses from a wide-ranging survey of human-wildlife conflicts and income sources in communities bordering the northern boundary of KNP, undertaken between the 12^th^ and 27^th^ of January 2024 (Fig 1). Secondly we studied 55 separate social media and online news portrayals of one incident in which an elephant left KNP and entered the bordering village of Matsulu, in the Mpumalanga District, South Africa on 10th February 2024 (Figs 1 and 2). We chose this event because it was recent, and captured on video by several members of the community and then extensively represented across multiple social media platforms, as well as news agencies (see Fig 2 for illustrative images).

### Community survey questionnaire

With respect to the community survey, a detailed methodology and further results are reported elsewhere (see [14]) but in brief we surveyed 12 communities located outside the northern boundary of KNP. Our study selected 1551 households, surveying household heads via a 40-question survey, administered face-to-face via mobile-based platform. between 16/01/2024 and 07/02/2024. The large geographic extent of some communities, and variations in their degree of dispersion, the quality of municipal records and types of settlements, made the implementation of a fully randomized sampling design financially and logistically prohibitive. Our sampling approach was designed to maintain methodological integrity while ensuring representativeness across settlement types. First a household sampling frame for each community was generated with the help of local authorities, listing household heads and verifying any unrecorded dwellings, which were subsequently added to the frame. For each community, a sampling interval (n) was calculated by dividing the total number of households by the required sample size, itself determined proportionally to the total sample allocation. Second, enumerators selected a random starting point and direction within each community. From this point, households were selected by applying the sampling interval (i.e., skipping n households between selections). If all households along a given direction were exhausted before reaching the required sample size, enumerators relocated to a new starting point and repeated the procedure. Enumerator teams were spatially separated to prevent overlap in sampling paths. Where a household head or other appropriate respondent was unavailable at the initial visit, enumerators were instructed to make up to two follow-up attempts at different times or days, after which if no suitable respondent was available the household was replaced following the sampling procedure. The total number of households approached and the number of refusals were not consistently recorded, and so are unavailable.

The survey instrument was designed following a desk review of literature, focus group discussions and key informant interviews with members of these communities to provide qualitative information that was used to refine the survey instrument. The full survey is available in Supplementary Materials. Within it we asked several questions specific to examining human-elephant conflict and the results of which are unreported elsewhere.

First we asked “How many different wild animal species have visited the community over the past five years?”, with respondents invited to answer by listing each species and the estimated number seen. For each species identified we asked, “Do members of the community ever encourage [the species] out from the park into the community?” and “Do members of the community ever block [the species] from exiting the community?”, with a binary yes/no response. These questions were asked because descriptions of the Matsulu incident implied that elephants had been encouraged from the park, and because initial focus-groups with local communities also suggested the occurrence of such practices.

Next we asked “Has anyone in the community been killed or injured by wildlife in the last five years?” with respondents who replied “yes” providing estimates of how many people were injured or killed over that period and to state which animals they believed were responsible. Such responses provide an index of the perceived likelihood of wildlife-related deaths and injuries among respondents, but were not used to estimate actual injury/death rates due to the high, but unmeasurable, likelihood of multiple respondents from the same community referring to the same individual events.

We then asked, “How do you feel about the lethal approach taken to deal with elephants entering the community?” with answers selected from a five-point Likert-type scale from “I strongly support it” to “I strongly don’t support it”. This question was followed by “Does the community receive any direct income or other benefits (e.g., meat or other body parts) when an elephant is killed in the community?” with a binary yes/no response. Respondents were then asked “Which benefits do the community receive when an elephant is killed?” with options of “money”, “meat”, “tusks” or “other”. Respondents who stated that the community received benefits were then asked “Do you think that this activity influences community members to encourage elephants out from the park?” and “Do you think that this behaviour influences members of the community to block elephants from exiting the community?”. Both follow-up questions had a binary yes/no response, and those answering “yes” were invited to explain why they felt that way (open responses).

Respondents were then asked “Do you personally receive any direct income when an elephant is killed in the community?” (yes/no response) with those who answered “yes” then asked “About how much did you [as an individual] receive in the last 12 months, in Rands?”. This question was followed by “Do you personally receive any other benefits when an elephant is killed in the community (e.g., meat or other body parts)?” (yes/no response) with those who answered “yes” asked, “How many kilograms of meat did you get in the last 12 months?”. The figures provided were directly recorded in their respective units to the nearest whole number.

Finally, respondents were asked “If there was an alternative non-lethal solution to elephants entering the community, would you support it?” (a binary yes/no response) and asked to explain why they felt that way (open responses).

The research was approved by the University of Pretoria’s Faculty of Natural and Agricultural Sciences Ethics Committee on 02.10.23, reference number NAS201/2023.

### Social media depictions of the Matsulu incident

With respect to the incident in Matsulu village, we initially searched for online portrayals using the search engine Google, using the search terms “elephant escape Kruger National Park”,

“elephant escape Matsulu South Africa” and “elephant escape Mpumalanga South Africa”. We then snowball sampled by locating similar videos identified by algorithms within a given social media platform. Searches and transcription of data were performed between 18/06/2024 and 22/11/2024. Criteria for inclusion were that the media were posted between 09/02/2024 and 01/06/2024, in any geographic location or language, to produce as large an initial set as possible (see Supplemental Materials). For each online portrayal we recorded the media platform [e.g., Facebook/TikTok/YouTube/Google etc], the channel type (e.g., Media, News, Personal, Influencer e.tc), the article description (copied directly from video), how the video/article describes what happened (whether in the audio, the video title/ brief summary, or in the body of a news article), what words or phrases were used to describe the elephant described in the video/article (e.g., “rampant”, “dangerous”, “wild”, “panicked”, “majestic”, “aggressive”, “beauty”), what words or phrases were used to describe the community (e.g., “angry mob”, “helpless community”, “bewildered”), what words or phrases are used to describe the authorities/ entity that kills the elephant (e.g., “harassed”, “overpowered”, “puzzled”, “threatened”), what drivers of the incident were mentioned in the video/article (e.g., “He asked the community not to plant mango and avocado trees near the fence that separates Matsulu and the KNP, as this could lure elephants and other wildlife out of the park”), and what solutions were mentioned in the video/article (e.g., “if everyone works together, they can save these animals for the sake of future generations”). All coding and attribution of media materials were checked by a second researcher, and any disagreements were settled through discussion.

### Statistical analyses

We wished to identify factors associated with support of non-lethal solutions, and with whether respondents had personally benefitted from the killing of elephants in their community (through obtaining money or meat). In both cases the variable was a binary yes/no variable, and analysed using ordinal logistic regressions (which are suitable for the analysis of ordinal data, including ordinal variables with two levels), implemented in Program R [46] using the ordinal package [47,48]. In each analysis available, relevant explanatory variables were respondents’ age and sex, the size of their household (number of individuals living in the house), their income, highest educational level, and distance of their community from the park fence. In the analysis of support for non-lethal solutions we additionally included as explanatory variables whether the respondent had stated there had been a death in the community from wildlife, and whether they had personally received a benefit from the killing of elephants (both binary yes/no variables *a priori* likely to affect attitudes to non-lethal control). Where appropriate, illustrative odds ratios were calculated from the regression coefficients for each variable factor of interest, as exp(coefficient) [48].

## Results

### Description and approximate timeline of the incident in Matsulu

On the 10th of February 2024, an elephant left KNP and entered the village of Matsulu in the South African province of Mpumalanga ([49], Fig 2). A complaint about the elephant was made to the MTPA (Mpumalanga Tourism and Parks Agency) from the Mandela Park community, and the MTPA dispatched their DCA (Damage Causing Animals) control unit [32] and helicopter support from KNP, with the intention of bringing the animal back to KNP [32,49]. Some previous such incidents of escaped elephants had been resolved with the return of the elephant to KNP, but a decision was made in this case to euthanise the elephant due to circumstances on the ground [32,50,51,49]. The local community was described as gathering around the elephant and throwing objects [32,49] [52] (Fig 2), as well as threatening MTPA members on the ground [53]. The DCA team was forced to temporarily withdraw, and the police (SAPS AND CPF) were called, but could not fully control the situation [53,52]. The DCA team then re-entered the community [32] and reportedly encouraged the elephant into an open field, where it was put down [32][52]. The head and tusks were removed and taken to an MTPA storage facility [32,51,52]. The community then butchered the elephant for its meat [32,50,53,49] using pangas, chainsaws and knives [50].

### How was the incident in Matsulu depicted in the media coverage?

We derived an inclusive list of 55 sources containing footage of the incident. Of these, 15 sources were secondary, comprising social media posts designed to highlight the original source in an alternative location, and linking to it (e.g., an X or Facebook post containing a link to an original news article, often by the organisation hosting that article), or constituted reposts of the exact same article by the same journalist on a different platform. Such secondary posts typically comprised a simple title and a media clip or a link to the primary source, and contained no information not available in the original, and we discounted such sources to prevent pseudoreplication. Of the remaining 40 primary sources, 35 were freely available to the public online, and five were available only to subscribers. To represent the breadth of interpretations of the incident in Matsulu while avoiding over-amplifying reposted articles and videos, we analysed only the 35 freely available primary sources.

Of the 35 primary sources 10 provided descriptions of the elephant in their text (see below for examples), of which eight sources were news articles and two were video descriptions. Similarly, 22 provided descriptions of the community in their text, of which 13 were news articles and nine were video descriptions. Three sources (two news articles, one video description) described the authorities, five (four articles, one video description) described drivers of the event, and seven (six articles, one video description) described possible solutions.

**Fig 3:**
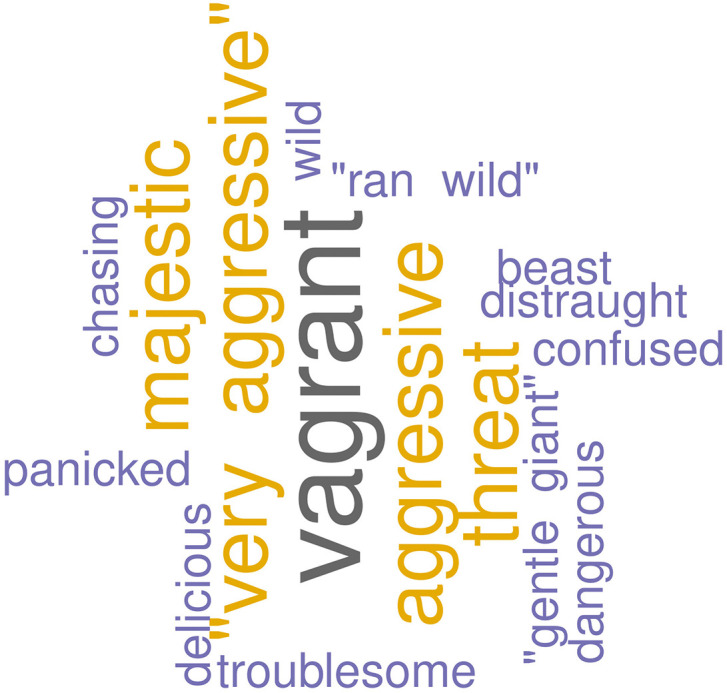
Words and phrases used to describe the elephant in 35 social media descriptions of the Matsulu incident (22 terms in total, largest word frequency = 3).

The 10 sources that described the elephant provided 16 individual terms describing the elephant (Fig 3). Of these six were used multiple times: “vagrant” (3); “aggressive” (2); “very aggressive” (2); “threat” (2); “majestic” (2), making a total of 22 usages of 16 terms (Fig 3). Ten usages (45.5% of all 22 used) were negative, indicating that the author of the text regarded the elephant’s behaviour as problematic (e.g., describing it as “dangerous”, “troublesome”, “aggressive”, or a “threat”). The remaining terms empathised the elephant’s behaviour (e.g., describing it as “confused”, “panicked”, “distraught”), were neutral (e.g., “vagrant”, “beast”) or were broadly positive (e.g., “gentle giant” and “majestic”), with one further article describing the elephant as “delicious”, referring to the meat the locals derived from its slaughter.

**Fig 4:**
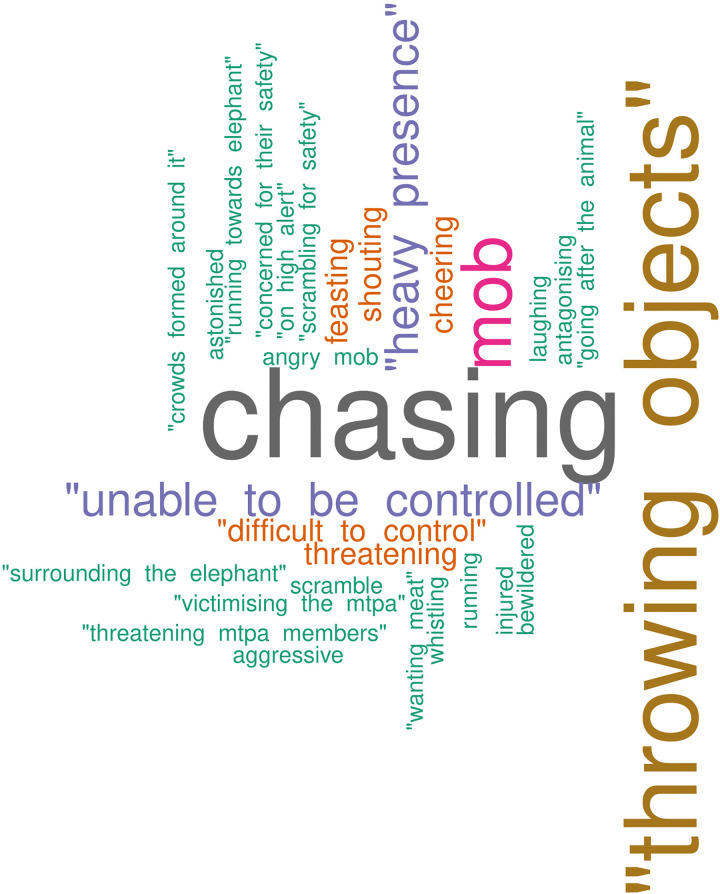
Words and phrases used to describe the community in 35 social media descriptions of the Matsulu incident (70 terms in total, largest word frequency = 14).

The 22 sources that provided descriptions of the community used 30 individual terms (Fig 4). Of these 11 were used multiple times: “chasing” (14), “throwing objects” (11); “mob” (7); “heavy presence” (4); “difficult to control” (2); “cheering” (2); “shouting” (2); “threatening” (2); “unable to be controlled” (2); “feasting” (2); “could not be controlled” (2) – making a total of 70 usages of 30 terms. Of the usages, 41 (58.6%) can be interpreted as directly critical of the community’s conduct (e.g., “unable to be controlled” “difficult to control”, “going after the animal”, “antagonising” “threatening MTPA members”), and a further 13 (18.6%) are implicitly critical (e.g., “mob”, “heavy presence”, “surrounding the elephant”). Eleven usages (15.7%) were broadly neutral (e.g., “cheering”, “shouting”, “laughing”, “feasting”, “on high alert”) but nonetheless many of these imply that the community to some extent welcomed the opportunity represented by the elephant. Only five usages (7.1%) suggested that the community suffered due to the elephants roaming freely outside protected areas(e.g., “injured” “bewildered” “scrambling for safety”). The authorities were described as being “astonished”, “victimised”, “overwhelmed” and “on high alert”.

Of the seven mentions of drivers of the event, four mentioned the planting of fruit trees near the KNP fence, one that fruits had been thrown into the park, one that the fence had been recently fixed and subsequently vandalised, and one mentioned that the community should not provoke or approach animals. One further mentioned that the elephant ruined the community’s property. Five sources mentioned that the community derived benefits in the form of meat from the slaughter of the elephant, but not in the context of this having been a driver of the event.

Of the seven solutions mentioned, four indicated that the community should report sightings and not approach animals, one asked the community to stay away from wildlife, one appealed to the community to respect law enforcement teams and one asked the community to help create initiatives to preserve elephants for future generations.

### Perceived factors affecting attitudes towards elephants in communities surrounding KNP

**Fig 5:**
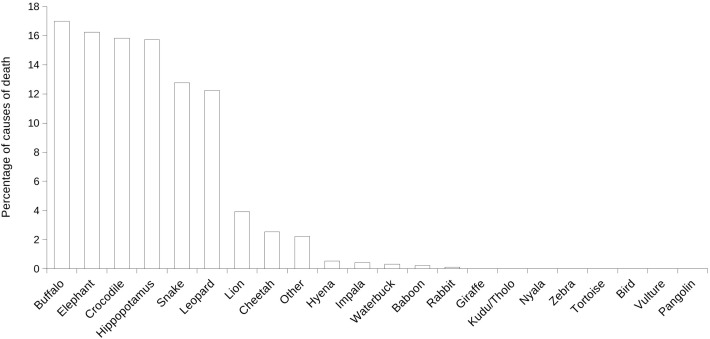
The percentage of human deaths in communities surrounding KNP attributed to different species of wildlife.

**Fig 6:**
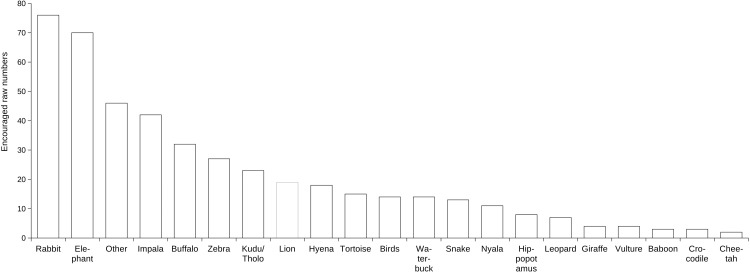
Raw numbers of mentions from our survey of each wildlife species being encouraged to leave KNP by members of neighbouring communities.

**Fig 7:**
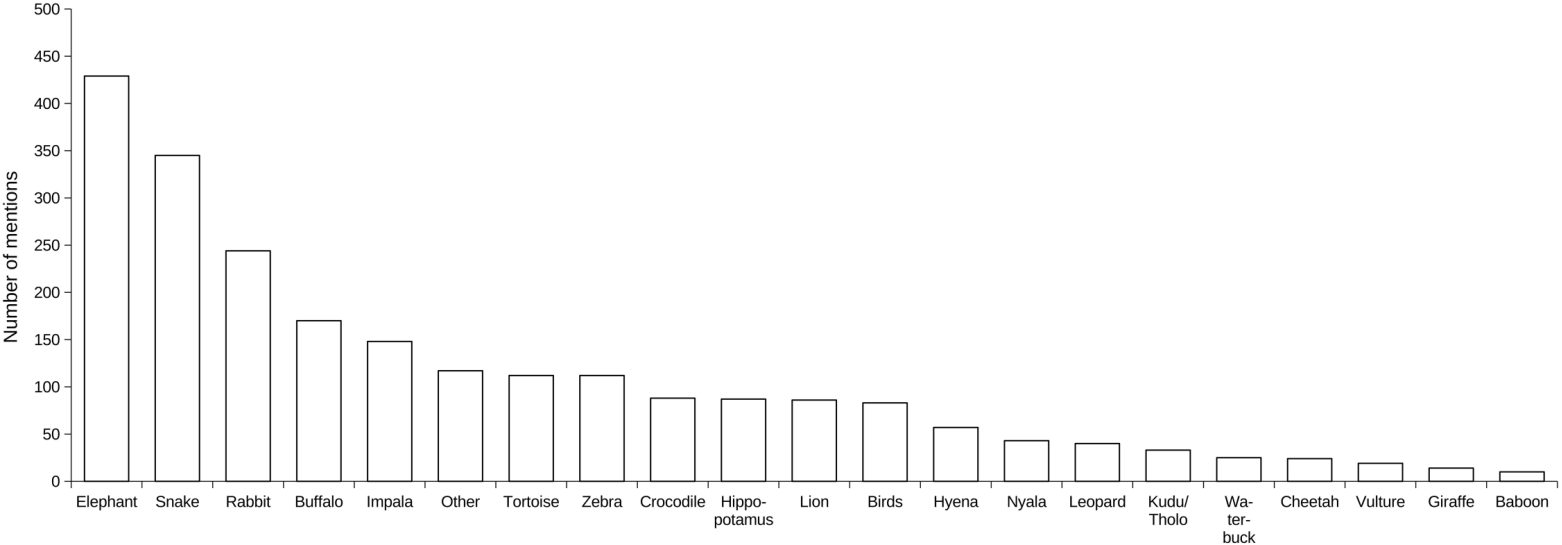
Raw numbers of mentions from our survey of each wildlife species being prevented from leaving the community, having entered.

When asked about wild-animal-related human deaths, 41.5% of respondents stated that at least one member of the community had been killed by wildlife in the last five years. When asked about the species responsible, 948 mentions of death were attributed across a range of different wildlife species. The most common species mentioned were buffalo (17.0% of all mentions), elephant (16.2%), crocodile (15.8%) and hippopotamus (15.7%) (Fig 5). It was not possible from our data to calculate the percentage of respondents directly affected by deaths of friends and relatives attributable to elephants, but 154 of 1551 (9.9%) respondents reported elephants as a cause of deaths in the community. When respondents were asked about the wildlife that had visited the community within the last five years, and whether they felt a given animal had been encouraged out of the park or blocked from leaving the village once arrived, it was clear that elephants were regarded as significant under each consideration. Elephants comprised 1067 mentions out of a total of 7157 mentions based on multiple responses (14.9% of all mentions) of species encountered in the community. The next most frequent species were snakes (884 mentions, 12.4%), baboons (492, 6.9%) and scrub hares (𝐿𝑒𝑝𝑢𝑠 𝑠𝑎𝑥𝑎𝑡𝑖𝑙𝑖𝑠; 479, 6.7%). In addition, Fig 6 demonstrates that elephants were one of the top two wildlife species most considered likely to be encouraged out of the park, with 76 mentions of rabbits, and 70 mentions of elephants being encouraged out. The percentage of respondents mentioning these animals, however, corresponds only to 4.9 and 4.5% of respondents believing that these animals are either deliberately encouraged into the village or visit communities in search of food. By comparison Fig 7 shows that elephants were the wildlife species considered most likely to be blocked from leaving the village, with 429 mentions overall, the next most blocked animals being snakes (325 mentions) and rabbits (244). In total 27.6% of respondents stated that elephants were typically deliberately blocked from leaving the community once arrived.

When respondents were asked if they personally received benefits when an elephant was killed in the community, 52.8% answered that they did, while 47.6% stated that they did not. These benefits were in the form of money (5.0% of the population, who stated that they had earned a mean of 8,535 rand – the equivalent of $457 – each in the last 12 months, in comparison to a mean monthly income of 4,027 rand among our survey respondents) and elephant meat (52.4% of the population stated that they had obtained a mean of 36.8 kg of meat each in the last 12 months).

Whether respondents received benefits received benefits from the killing of elephants was associated with their sex, income and the distance of their community to the park border, in models also incorporating respondents’ age, education and household size (Table 1). Odds ratios revealed that male respondents were 1.2 times more likely to state that they received benefits than were female respondents. Respondents with the highest annual incomes were 3.1 times less likely to receive benefits from killing elephants than were those with the lowest incomes. Respondents living at the greatest distances from the park fence (>70km) were 19.0 times less likely to state that they had received benefits from the killing of elephants than were those immediately adjacent to the border.

**Table 1:** Likelihood ratio tests of factors associated with whether a respondent stated they received benefits (money or meat) from the killing of elephants.

Source	D.F.	LRT	P
Age	1	0.0008	0.977
Sex	1	3.91	0.0480
‍Education	1	0.842	0.359
‍Household size	1	1.61	0.204
‍Income	1	4.47	0.0346
‍Distance to park border	1	21.375	<0.001

When asked if they thought that these above benefits might encourage members of the community to encourage elephants to leave KNP, 11.9% of respondents replied “Yes”. And when asked if they thought that these benefits might influence members of the community to block elephants from exiting the community (once the elephant had entered), 39.4% of respondents replied “Yes”.

### Evidence for reasons for encouraging elephants from the park, blocking elephants and supporting lethal control

The 11.9% (n = 144) of respondents who thought that benefits might incite members of the community to encourage elephants from the park were asked why they felt that way. Of the resulting 139 interpretable answers from these respondents, 68.3% were that meat/ food from the slaughter of elephants were the reason (e.g., “To get meat and feed our families”, “So they can have meat and money”), while a non-mutually-exclusive 11.5% stated the money gained was a prime motivation (e.g., “Because we need money to live and this is the only way of living”). An additional 15.1% of responses mentioned that the fence of KNP had been deliberately vandalised to encourage elephants out (e.g., “We have poachers sometimes who damaged the fence so animals find a way of coming out and being killed, when it killed people benefit”; “Because fences are broken by hunters from the community”).

The 39.4% (n = 476) of respondents who stated that the benefits incited community members to deliberately block elephants from leaving the community yielded 427 interpretable responses when asked why they felt that way. Of these responses, 76.1% were that meat and/or food from slaughtered elephants were a prime motivator (e.g., “Because we get meat by blocking them in and killing them”), and 14.3% of responses stated that money was a motivator (e.g., “Because people get meat and the community will get money to buy things that are needed in the community”; “Because people know that they sometimes get meat or money when an elephants is killed”). In addition, 18.5% of responses mentioned that elephants were a danger to humans, and the community needed protecting (e.g., “Because people feel like if they don’t kill the elephants, the elephants will kill the people”; “Because they have killed our livestock and our relatives”). A further 2.1% mentioned the necessity of protecting crops and livestock (e.g., “Elephants will kill human beings and destroy our crops”), and 0.7% made reference to a belief that elephants outside of the park belonged to the community (e.g., “Because once it’s outside of the Kruger national park, it’s ours and we must kill it before it kills us” and “We know that if the elephant enters the community it is ours”).

### Attitudes towards alternative elephant management approaches

When asked in our survey about how strongly they supported the lethal control of elephants, 61.7% (n = 1208) of respondents voiced support, with (51.8% “strongly” supporting it and 9.9% “somewhat” supporting it. By comparison 33.9% (n = 303) stated that they did not support the control, with 29.9% “strongly” not supporting it.

When, however, respondents were asked whether if there was an alternative, non-lethal solution to elephants entering the community, would they support it, 72.1% (n = 1117) indicated that they would. When asked to explain their response, the 72.1% who indicated they would support non-lethal solutions yielded 1047 interpretable responses. Of these, 51.1% indicated that their preference was predicated on species conservation and/or animal protection (e.g., “they play an important role in the ecosystem” and “they should live so the number of elephants can increase”). A further 20.5% of responses indicated that they wished to see elephants preserved for future generations (e.g., “elephants need to be protected for future generations”). Another 15.7% of respondents stated that non-lethal control methods would protect human lives and livelihoods within the community (e.g., “Because the community will be safe”; “Because our crops will be safe from the elephant”), and 9.1% that the existence of elephants provided a source of income (e.g., “Because elephants have value, we gain money from elephants without killing them”). Finally 5.5% of respondents indicated that elephants had an intrinsic right to exist (e.g., “Because the elephant is like a human being - it must live”), 5.2% that elephants promoted tourism (e.g., “Elephants play a big role in tourism”), 2.5% that elephants were part of the community’s heritage, and <1% that slaughtering and cutting up elephants for meat was a source of accidental injuries.

When asked to explain their reply the 27.9% (n = 434) who stated that they would not support non-lethal solutions yielded 327 interpretable responses. These were divided between 42.8% that referenced benefits from lethally controlling elephants, and 52.3% that referenced disbenefits of the presence of elephants. The benefits comprised – non-exclusively – 29.7% of replies referencing meat and food, 2.8% referencing money/ income (e.g., “Because the meat and the money which the community gets, help a lot”) and 15.6% referencing unspecified benefits (e.g., “Because we will not be benefiting”). The disbenefits comprised 46.8% citing direct threats to humans, and a further non-exclusive 12.7% citing threats to crops and/or livelihoods (e.g., “Because those elephant is destroying our crop and threatening our lives so they have to be killed”). An additional 1.5% of responses stated that elephants, once out of Kruger Park, belong to the community (e.g., “Because according to the Kruger national park law, once an animal is out of the park it belongs to the community”). And finally 3.1% stated that the elephants would keep returning once they had discovered the community (e.g., “The same elephants will keep coming back but getting rid of it brings peace to locals”).

Support for non-lethal control of elephants varied with respondents’ sex, household size, whether they perceived there to have been deaths in the community due to wildlife, whether they had previous received personal benefits (money or meat) from the killing of elephants, and with distance to the KNP border (Table 2). Odds ratios revealed that male respondents were 1.4 times less likely support non-lethal control than were female respondents. Similarly, respondents with the largest households (17 members) were 3.0 times less likely to support non-lethal control than were those with single occupancy households. Respondents who stated they know people who had been killed or injured by wildlife in the last five years were 1.4 times less likely to support non-lethal control, and those who had received personal benefits – either money or meat – from the killing of elephants were 1.5 times less likely to support non-lethal control. Respondents in communities at the furthest distance from KNP were 4.6 times more likely to support non-lethal control than those directly adjacent to the park.

**Table 2:** Likelihood ratio tests of factors affecting support for non-lethal elephant control in communities adjacent to KNP.

Source	D.F.	LRT	P
Age	1	0.0026	>0.959
Sex	1	7.74	0.00539
Distance to park border	1	7.91	0.00492
Education	1	3.56	0.0592
Household size	1	6.30	0.0120
Income	1	0.619	0.431
Wildlife death in community?	1	8.79	0.003 03
Personal benefit received from killing elephant?	1	12.9	<0.001

## Discussion

A majority of respondents to our survey of members of communities bordering KNP (52.8%, n = 812) stated that they received benefits from the slaughter of visiting elephants – a species regarded as frequent visitors to those communities – typically in the form of meat. A substantially smaller proportion of respondents (5.0%) stated that they benefitted in pecuniary terms. The benefits from the slaughter of elephants (both meat and financial) were considered by well over a third of respondents (39.4%) to be a key stimulus that encouraged people to block elephants from exiting the community once they had arrived. A smaller proportion of respondents (11.9%) felt that the benefits incited community members to encourage elephants to leave the park. This finding accords with the suggestion made by a number of respondents that specific groups (described as “hunters” or “poachers” by survey respondents) have been responsible for damaging the park’s fence, which can permit the egress of elephants.

While it cannot be ascertained conclusively, taken together all the above evidence suggests that events such as the Matsulu incident may be initiated by the actions of a relatively small number of individuals, through damaging the KNP park fence, and encouraging elephants to exit the park. The mention by respondents in our surveys, and in media descriptions of the events in Matsulu, that the KNP fence had been deliberately vandalised by community members (specifically poachers) and that fruit trees had been planted next to the park fence, and fruit thrown into the park (noted in several social media descriptions of the Matsulu incident) suggests deliberate attempts to entice elephants from the park. The principal benefits of doing so appear to accrue to a small number of individuals who benefit financially, and relatively substantially, representing two months of pay at average income for the population.

The wider community appears to experience a balance of benefits and costs when elephants leave the park and enter the village and surrounding area. Reasons for the majority support of lethal elephant control (61.7% of respondents supported it) are likely to stem at least in part from costs from the presence of elephants in proximity to the community, which take the form of human deaths – approximately 10% of respondents reported knowing someone who had been killed by elephants in the last five years – and threats to crops and livelihoods (mentioned by 12.7% of those opposed to non-lethal elephant control strategies). These figures are unlikely to accurately represent the actual numbers of injuries and deaths due to the likelihood of multiple respondents mentioning the same events, but nonetheless many members of the community clearly perceive that they have suffered losses resulting from elephants escaping, or being encouraged or permitted to leave, KNP. It is equally clear that some community members also value the financial and food benefits deriving from the slaughter of elephants that enter the community, and our analyses demonstrate that such benefits accrue most to community members who have smaller incomes and who live closer to the KNP border. Overall, however, approximately three quarters of the community members surveyed expressed a desire for elephants to be managed via non-lethal control methods. Key correlates of support for non-lethal control were whether respondents stated they knew of someone killed/ injured by wildlife and whether they had experienced benefits from the slaughter of elephants, both of which made support for non-lethal control less likely, and distance from the KNP border, which made support more likely. Taken together these findings indicate broad, majority support for not killing elephants, despite the benefits of doing so – if non-lethal management resulted in the community experiencing less conflict – but highlight that such attitudes were also negatively affected by both perceived benefits from lethal control and perceived risks from wildlife. We speculate that the positive correlation between distance from the KNP border and support for non-lethal control represents autocorrelation between the perception/ experience of these risks and benefits, and distance.

In summary it appears likely that events such as the Matsulu incident may plausibly be perpetuated by a small number of actors in the community, for financial gain, with the wider community experiencing sufficient benefits (e.g., in the form of meat) for those actions to be tolerated, even if the community in general might prefer such conflicts not to occur, and/or experience losses as a result. We draw this inference acknowledging that it relies on recorded perceptions rather than observed behaviour, and that a number of alternative explanations (e.g., fence maintenance cycles, seasonal forage availability, water points) might exist for elephants leaving KNP and entering communities. Nevertheless, our observations raise the prospect that coexistence with elephants would be feasible if the wider community were to derive benefits from doing so, e.g., through non-consumptive wildlife-based livelihoods (see, e.g., [14]).

The above narrative was partially represented in social media commentary on the Matsulu incident. Of the 41 terms used to describe the community, 58.6% could be interpreted as directly critical of the community’s conduct, and a further 18.6% were implicitly critical. The typical narrative represented the community’s behaviour as crowding around, pursuing the elephant and impeding members of the MTPA and law enforcement officers who were attempting to resolve the situation, thereby ultimately leading to the elephant’s death. Descriptions of the elephant were less negative, with 45.5% describing it in negative terms (e.g., as being “dangerous” or “aggressive”), with the remainder either neutral or positive.

Where perceived drivers of the Matsulu incident were reported (in seven of 22 relevant media sources), the majority (six) implied that the community played a role, by planting fruit trees close to the park fence, throwing fruit into the park, vandalising the fence, and crowding around and pursuing escaped elephants. Only one article explicitly suggested that the presence of elephants *per se* could be an underlying cause of problems for the community: this article stated that elephants ruined the community’s property, adding that the residents threatened to strike and close the roads because the issue was not being addressed [54]. No articles, however, mentioned the potential financial benefits to community members from the killing of elephants.

We conclude that overall the narrative in the media coverage highlighted many of the same drivers of elephant killings that we derived from the survey responses: that members of the community may have tampered with the KNP fence, encouraged elephants to exit the park and then crowded around the elephant, resulting in its having to be killed, with the community then gaining access to meat. Missing from media narratives, however, was the observation that a small number of community members stand to profit financially from elephant escapes, potentially an important driver of human-elephant conflict in the area.

Due to the non-experimental nature of our data, we were unable to demonstrate conclusively that members of local communities were incentivised to vandalise the KNP fence in the expectation of achieving monetary or non-monetary rewards. Our data were sufficient to demonstrate that both survey respondents and social media descriptions of the Matsulu incident believed that local communities have vandalised the KNP fence, and that a minority of individuals from local communities profit financially from the slaughter of elephants, as well as the majority benefitting from obtaining meat. We have no way of knowing, however, if the individuals profiting the most were in any way linked to those destroying the fence. Nevertheless our findings are consistent with the possibility that elephant escape incidents, such as the incident that occurred in Matsulu, are being deliberately encouraged by community members, and that the most probable motive for doing so would be anticipated benefits in the form of money and/or meat.

## Conclusions and recommendations

The current policy [28] regarding elephants in KNP is that elephants are protected within the park but when approaching human communities are considered to be potentially “damage causing animals”, and if considered a danger to human life are permitted to be killed. This policy has clear implications for elephant conservation in a context where members of local communities could feel incentivised to facilitate the escape of elephants from KNP, in order to legitimately kill them. Accordingly we recommend an evaluation of the impacts of this policy in the light of community attitudes towards elephant conflict. Such evaluations should involve detailed discussions with communities, bringing together a range of other stakeholders to get a diversity of perspectives, particularly because the majority of the respondents in our survey expressed a desire for non-lethal control of elephants, largely motivated by a desire to protect and conserve elephants, to preserve wild animals for future generations, and to avoid losses of life and livelihoods from human-elephant conflict within communities. Our findings are consistent with the conclusion that the lethal approach to elephant management might encourage a proportion of community members to take a consumptive attitude towards elephants, which in turn may increase the likelihood of the community in general experiencing losses of life and livelihood due to human-elephant encounters.

A significant proportion of the individuals surveyed stated they were willing to embrace non-lethal management of elephants, for reasons that include saving them for future generations. This raises the possibility that the introduction of alternative livelihoods based on non-consumptive use of wildlife could significantly ameliorate future conflict. Elephants are sentient, charismatic animals that are a draw for tourists in South Africa and an important constituent of the tourism economy [55]. Previous work has shown that communities local to KNP would welcome non-consumptive income streams [14], and so in the light of findings from the current study we recommend reconsideration of the current policy concerning wild animals outside of KNP, with a view to prioritising non-lethal approaches to elephant management.
